# A Rare Presentation of Primary Central Nervous System Lymphoma in an Immunocompetent Patient

**DOI:** 10.7759/cureus.23858

**Published:** 2022-04-05

**Authors:** Nathan DeRon, Maheen Ahmed, Dylan Lopez, Ahmed Alobaidi

**Affiliations:** 1 Internal Medicine, Methodist Health System, Dallas, USA; 2 Internal Medicine, Texas College of Osteopathic Medicine, Fort Worth, USA

**Keywords:** ataxia, stroke, case report, lymphoma treatment, lymphoma management, lymphoma in immunocompetent, lymphoma, cns lymphoma, primary cns lymphoma

## Abstract

Primary central nervous system lymphoma (PCNSL) is a rare non-Hodgkin's lymphoma (NHL) that can develop in the brain, spinal cord, leptomeninges, and vitreoretinal space. The majority of cases are diffuse large B-cell lymphomas. Risk factors include immune dysfunction, prior Epstein-Barr viral infection, HIV, and a family history of non-Hodgkin's lymphoma. Although the majority of the patients are immunocompromised, PCNSL is still seen in immunocompetent patients. PCNSL has a poor prognosis and a high relapse rate despite its radiosensitive and chemosensitive nature. It is important to recognize and diagnose PCNSL early to improve outcomes. We present a case of PCNSL in an immunocompetent adult with no previously known risk factors.

We present a case of a 66-year-old male who presented with a 1.5-week history of right-sided headache and left-sided weakness. After being admitted for further evaluation, he underwent multiple laboratory tests and imaging studies. The CT head indicated ill-defined hypodensities in the pons and left cerebellum. CTA revealed a 1.5 cm outpouching along the medial aspect of the distal left cervical internal carotid artery at the C1-C2 level concerning a pseudoaneurysm. Neurology was consulted, and an MRI of the brain revealed equivocal brain lesions. Neurosurgery was consulted, and the patient underwent an open brain biopsy, which revealed a high likelihood of primary CNS lymphoma based on intraoperative pathology findings. CSF analysis revealed an elevated percentage of lymphocytes, including the presence of atypical lymphocytes as well as elevated oligoclonal bands. Subsequent pathology results confirmed PCNSL. The oncology service was consulted, and the patient was started on corticosteroids and methotrexate for chemotherapy as well as leucovorin.

This case represents a rare presentation of PCNSL in which the patient had no known history to support an immunocompromised state. Imaging findings, in this case, were also atypical for a primary CNS lesion as they were mostly equivocal. Furthermore, imaging findings showed diffuse CNS disease rather than an obvious primary lesion as typically demonstrated in the literature. In this case, the open brain biopsy was pivotal in making a timely diagnosis and beginning disease-modifying therapy as early as possible. This case demonstrates the imperative need for clinicians to be aware of varying presentations of PCNSL and possibly consider pursuing a definitive diagnosis with biopsy when the differential includes PCNSL but remains broad after advanced imaging.

## Introduction

Lymphoma is a neoplastic proliferation of lymphoid cells that forms a mass arising from lymph nodes or extranodal lymphatic tissue. It is classified into two subtypes, including Hodgkin's and non-Hodgkin's lymphoma (NHL). Primary CNS lymphoma is a group of rare and aggressive NHLs that account for 2-3% of all brain tumors [1], and 90% of all PCNSLs are ultimately found to be diffuse large B-cell lymphomas [2]. CNS lymphoma only affects approximately 1600 people per year in the United States, and the median age at diagnosis is 67 years [3]. The overall incidence of PCNSL in immunocompromised patients has been decreasing since the 1990s [4]. However, the incidence has been increasing in immunocompetent patients, especially in adults older than 65 years of age [5]. Clinical presentations often include patients developing neurologic signs over weeks, which include personality changes, changes in speech, focal neurological deficits, symptoms of increased intracranial pressure, seizures, muscle weakness, ataxia, vomiting, confusion, and vision changes [6]. Based on the wide variety of symptoms and the increasing incidence in immunocompetent patients, it is important to review the variable presentations in immunocompetent patients with PCNSL to improve the alacrity of diagnostic recognition and to begin early appropriate targeted therapy.

## Case presentation

The patient is a 66-year-old male with a history of nephrolithiasis and insomnia presenting with a right-sided headache and left-sided weakness for 1.5 weeks. The patient had begun to lean to the left while walking and even suffered an abrasion to his left arm on a structure in his home due to this imbalance. The patient reported an unintentional weight loss of 60 pounds over the previous six months but simply attributed it to a decreased appetite. The patient denied any prior trauma or additional symptoms such as shortness of breath, chest pain, or genitourinary complaints. The patient reported no significant family medical history. Initial physical exams revealed significant dysarthria, 4/5 strength in the left upper and lower extremities, and bilateral dysconjugate gaze. Due to this presentation, the patient immediately underwent stroke imaging and work-up protocol in the emergency department. The transthoracic echo, the lipid panel, and HbA1c were all found to be within normal limits. However, CT head imaging indicated ill-defined hypodensities in the right pons and left cerebellum. A CTA head revealed a 1.5-centimeter outpouching along the medial aspect of the distal left cervical internal carotid artery at the C1-C2 level, concerning a pseudoaneurysm. The neurology service was consulted and recommended further lab work, including serum HIV, vitamin B12 level, folate level, and serum RPR, as well as an MRI of the brain. Outpatient follow-up was also recommended for the pseudoaneurysm, which appeared to be clinically irrelevant. Vitamin B12 and folate levels, as well as RPR titers, were found to be within normal limits. Serum HIV was found to be negative. The patient then underwent an MRI of the brain which revealed multiple abnormal areas on T2 FLAIR, including in the left thalamus, left anterior limb of the internal capsule, left basal ganglia, right inferior thalamus and midbrain, right paramedian pons, left cerebellar hemisphere and vermis, and right middle cerebellar peduncle, as exhibited below.

**Figure 1:**
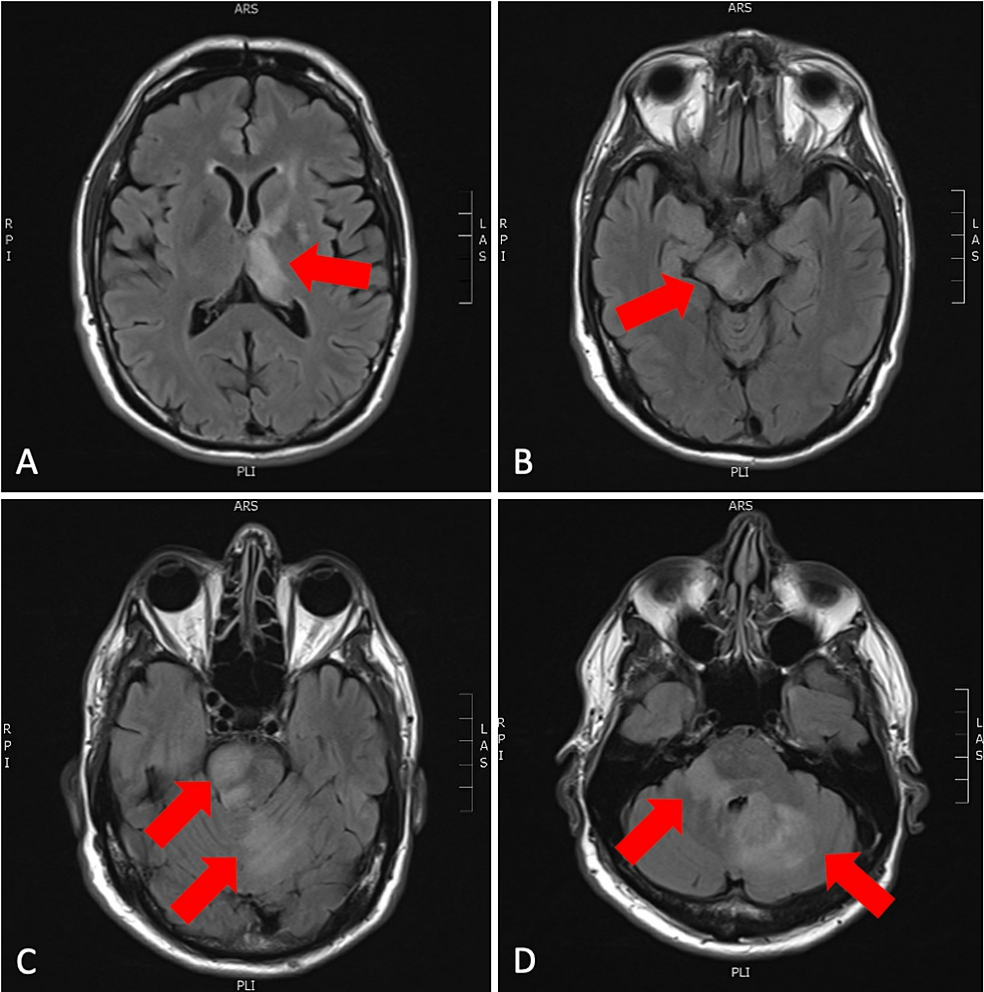
Axial slices from T2 FLAIR sequence of MRI brain with and without contrast. (A) Left thalamus and internal capsule involvement; (B) right midbrain involvement; (C) right pons and left cerebellar involvement; (D) bilateral cerebellar involvement.

The results of the MRI were not consistent with a subacute stroke, and based on the images, the differential included, but was not limited to, an acute demyelinating disorder, encephalitis with a possible viral origin, and a primary neoplastic process such as glioma or lymphoma. Per neurology, and based on the history of the present illness and the multiple equivocal enhancing brain lesions found on MRI, there was a high likelihood of CNS lymphoma. For a definitive diagnosis, neurology recommended a brain biopsy and lumbar puncture. The neurosurgery service was consulted, and the patient underwent craniotomy with an open brain biopsy of the cerebellar lesions. Intraoperative pathology was reportedly favored to be primary CNS lymphoma. CSF analysis from lumbar puncture revealed an elevated percentage of lymphocytes, including the presence of atypical lymphocytes as well as elevated oligoclonal bands. Definitive pathology results revealed diffuse large B-cell lymphoma negative for MYC translocation and BCL2 and BCL6 gene rearrangements. Pathology slide images are pictured below, along with a table of flow cytometry marker findings and interpretations (Table 1).

**Figure 2:**
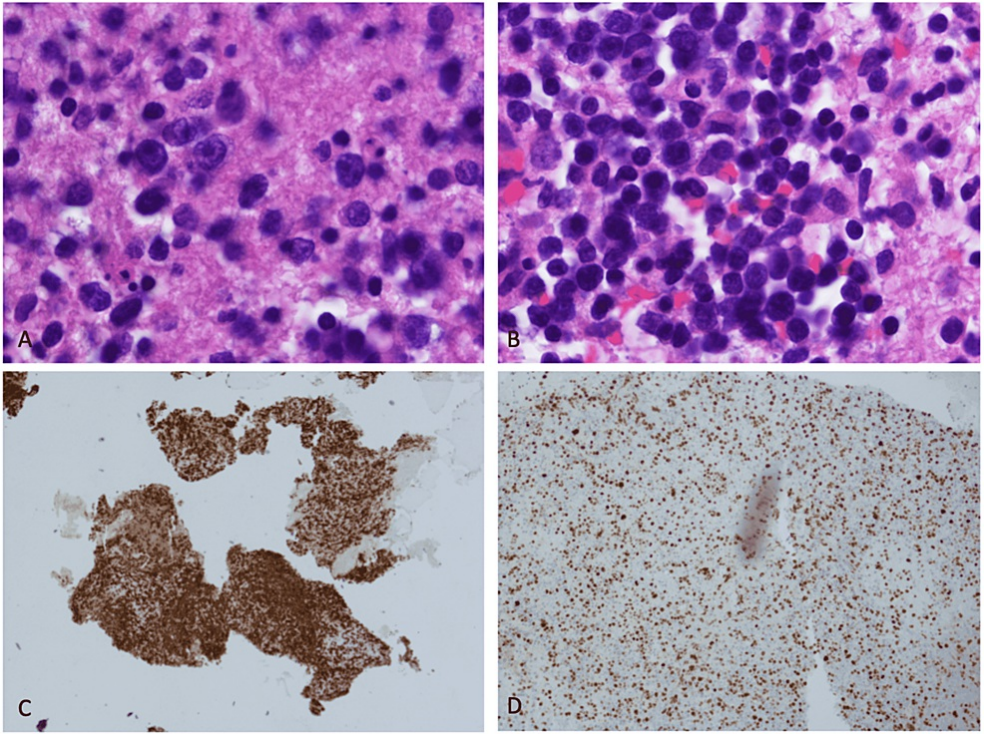
Images of stained pathology slides from brain lesion biopsy. (A) H&E illustrating darkly stained tumor cells; (B) additional H&E of tumor cells; (C) CD20 stain suggestive of increased B lymphocytes; and (D) Ki-67 stain indicating increased cell proliferation.

**Table 1:** Definitive pathology findings from brain biopsy and associated interpretation of findings.

Marker	Results	Interpretation
CD 5	Negative	Decreased likelihood of T-cell lymphoma and chronic B-cell leukemia
CD 10	Negative	Decreased likelihood of acute leukemia and follicular lymphoma
CD 19	Positive	Highly suggestive of B-cell malignancy and available target for CAR-T and monoclonal antibody medications
CD 20	Positive	Suggests active B-cells; expressed in B-cell lymphomas
CD 23	Negative	Decreased likelihood B-cells involved in capturing IgE-antigen complexes
CD 38	Equivocal	Decreased likelihood that HIV seroconversion is occurring
CD 45	Positive	Increased likelihood of B-cell leukemia or lymphoma
FMC7	Positive	Strong likelihood of B-cell NHL or chronic lymphocytic leukemia [7]
Additional findings	Aberrant population (<1%) of lambda light chain restricted	Highly suggestive of malignant B-cell proliferation and movement away from normal kappa/lambda ratio of 1:1 to 1:2 [8]

Subsequent serum Epstein-Barr virus (EBV) IgG levels were obtained and noted to be elevated at an index greater than 5.5 units, indicating that past EBV infection was now resolved. The oncology service was consulted, and the patient was started on intravenous corticosteroids, high-dose methotrexate, and leucovorin. A testicular ultrasound was obtained and was negative for the neoplastic process. CT chest/abdomen/pelvis with contrast was negative for metastatic disease. Methotrexate levels were monitored daily and declined as expected. The patient was discharged in stable condition. Follow-up was arranged with hematology/oncology, neurology, and neurosurgery to monitor responsiveness to chemotherapy.

## Discussion

Establishing the diagnosis of primary CNS lymphoma is challenging due to its varying clinical presentations. The most common symptoms include cognitive decline and gait disturbance, but the differential diagnosis in patients presenting with these symptoms is vast [2]. Some studies even show focal neurological deficit and behavioral disturbance as common presentations of PCNSL [9]. The patient in this case was found to have ataxia but revealed no signs of declining mental status or recent personality changes. Given this patient’s presentation with left-sided weakness and right-sided headache combined with the findings on the CT head, the patient was initially thought to have experienced a stroke. However, the MRI brain revealed multiple enhancing lesions, which prompted the subsequent workup for a neoplastic process, ultimately resulting in the finding of PCNSL. Full staging work-up for PCNSL includes MRI of the brain and spine, lumbar puncture, PET scan or CT chest/abdomen/pelvis with IV contrast, bone marrow biopsy, and a testicular ultrasound [10].

Studies have shown that PCNSL often presents with homogeneously enhancing deep brain lesions on MRI [2]. The characteristic appearance of PCNSL on T1 weighted MRIs is an isointense or hypointense mass, and an isointense or hyperintense mass on T2-weighted MRIs [2]. A ring-enhancing pattern is often seen in immunocompromised patients. In immunocompetent patients, MRIs often show a solitary, homogeneous enhancing mass in the parenchyma. In approximately 40% of the cases with immunocompetent patients, multiple lesions were seen on MRI [4].

The gold standard for diagnosing CNS lymphoma is a stereotactic biopsy, which was performed in this case. It is important to note that steroids should not be administered prior to the biopsy since they may rapidly improve radiographic features and make the biopsy more technically difficult while also skewing pathology results [2]. The patient in this case also demonstrated elevated oligoclonal bands in the CSF analysis, although the IgG index was within normal limits. There is limited evidence that elevated oligoclonal bands may be suggestive of PCNSL [11]; however, these data are not conclusive, and the presence of oligoclonal bands may be seen in other disease processes such as multiple sclerosis.

Additionally, patients diagnosed with PCNSL are at a significantly increased risk for venous thromboembolism, as noted in multiple retrospective analyses of this patient population. One study noted an incidence of greater than 50% of patients with positive venous thromboembolism (VTE) finding, of which 7% proved fatal [12]. This risk is significant and proffers the question regarding the risk balance between VTE prophylaxis administration and the increased risk of intralesional hemorrhage. Suggestions have been made that the mortality benefit from starting VTE prophylaxis with low-molecular-weight heparin likely outweighs the increased risk of spontaneous intralesional hemorrhage, but this treatment strategy continues to be in the data-gathering phase without a definitive recommendation [13].

For both immunocompetent and HIV-related PCNSL, the field has moved increasingly away from whole-brain radiation therapy as first-line or consolidative therapy due to poor durability of responses, poor survival when used as a standalone therapy, and risks of irreversible neurocognitive decline in survivors [14]. The field now appears to mostly employ high-dose methotrexate combined with corticosteroids as first-line therapy for PCNSL. According to new literature, patients receiving high-dose methotrexate alone exhibited non-inferior survival outcomes with decreased neurotoxicity versus whole brain radiation therapy [15]. However, there are many ongoing studies investigating alternative chemotherapy medications for PCNSL. Anti-PD-1 agents such as nivolumab and pembrolizumab appear to show promising results with regard to complete response and improved clinical outcomes. CAR-T appears to also show preliminarily promising results when crafted to target CD19 [16]. These results are limited, though, due to their minimal literature support and use mostly in refractory PCNSL cases given the proven effectiveness of high-dose methotrexate.

Discussion regarding whether surgery has a role in PCNSL is a complex issue with multiple variable factors. For patients with a large primary lesion as the prominent tumor burden, surgery may be considered to improve quality of life, although prolonged survival continues to be in question [17]. The clinical decision to pursue a debulking surgical procedure should include multiple factors such as age, performance status, and deep brain structure involvement [18]. However, in cases in which the tumor burden is diffuse and affects multiple neurologic structures, surgery may be impractical.

## Conclusions

Due to the low incidence of primary CNS lymphoma and nonspecific symptoms in immunocompetent individuals, this diagnosis is often overlooked. Based on the wide range of presentations of PCNSL and given the disease's proclivity to affect multiple nonadjacent areas of the brain, it is important to understand the different clinical manifestations of the disease for improved recognition of the disease. In addition, it is imperative to recognize that imaging findings vary widely, as evidenced in this patient who did not have a solitary lesion but a more diffuse CNS involvement pattern. Traditionally, PCNSL was known as an AIDS-defining illness. However, as exhibited by the above-described case and the literature reviewed thereafter, we believe this definition continues to become obsolete. It is imperative that clinicians recognize the variable clinical presentations of PCNSL and, when appropriate, consider this diagnosis in the differential to improve the alacrity of diagnosis and thus decrease the time to initial treatment for better overall clinical outcomes.
